# Transport, ultrastructural localization, and distribution of chemical forms of lead in radish (*Raphanus sativus* L.)

**DOI:** 10.3389/fpls.2015.00293

**Published:** 2015-05-08

**Authors:** Yan Wang, Hong Shen, Liang Xu, Xianwen Zhu, Chao Li, Wei Zhang, Yang Xie, Yiqin Gong, Liwang Liu

**Affiliations:** ^1^National Key Laboratory of Crop Genetics and Germplasm Enhancement, College of Horticulture, Nanjing Agricultural UniversityNanjing, China; ^2^Department of Plant Sciences, North Dakota State UniversityFargo, ND, USA

**Keywords:** radish (*Raphanus sativus* L.), lead, chemical form, subcellular distribution, translocation

## Abstract

Lead (Pb), a ubiquitous but highly toxic heavy metal (HM), is harmful to human health through various pathways including by ingestion of contaminated vegetables. Radish is a worldwide root vegetable crop with significant health and nutritional benefits. However, little is known about Pb translocation and distribution within radish plants after its uptake by the roots. In this study, Pb stress was induced using Pb(NO_3_)_2_ in hydroponic culture, aiming to characterize the transport, ultrastructural localization, and distribution of chemical forms of Pb in different tissues of radish. The results showed that the majority of Pb (85.76–98.72%) was retained in underground organs including lateral roots, root heads and taproot skins, while a small proportion of Pb was absorbed by root flesh (0.44–1.56%) or transported to the shoot (1.28–14.24%). A large proportion of Pb (74.11–99.30%) was integrated with undissolved Pb oxalate, protein and pectates forming Pb–phosphate complexes. Moreover, a low-Pb-accumulating line of radish showed a higher proportion of Pb in water-soluble form compared with a high-Pb-accumulating line. Subcellular distribution analysis showed that a large proportion of Pb was bound to cell wall fraction in lateral roots (71.08–80.40%) and taproot skin (46.22–77.94%), while the leaves and roots had 28.36–39.37% and 27.35–46.51% of Pb stored in the soluble fraction, respectively. Furthermore, transmission electron microscopy (TEM) revealed Pb precipitates in intercellular space, cell wall, plasma lemma and vacuoles. Fractionation results also showed the accumulation of Pb on the cell wall, intercellular space and vacuole, and low uptake of undissolved Pb oxalate, protein, pectates and Pb–phosphate complexes, which might be due to low transport efficiency and Pb tolerance of radish. These findings would provide insight into molecular mechanism of Pb uptake and translocation in radish and facilitate development of low-Pb-content cultivars in root vegetable crops.

## Introduction

Contamination by heavy metals (HMs) has become a worldwide environmental problem, because the metals easily accumulate in soil and are difficult to volatilize, dissolve or be decomposed by soil microorganisms (Cui and Zhang, 2004; Abou-Shanab et al., 2007; Zhang et al., 2012). Lead (Pb) is one of the five most toxic HMs and is harmful to human beings and other creatures (Wang et al., 2013). Other than natural weathering processes, the main source of contamination of agricultural soils and water by Pb is usually a direct or indirect consequence of anthropogenic activities (McLaughlin and Singh, 1999; Sharma and Dubey, 2005). The sources of anthropogenic contamination include Pb-using industries such as battery manufacture, metal mining and smelting (Capdevila et al., 2003; Caussy et al., 2003), urban and industrial wastes (Singh, 2001), fumes of automobiles and industry, fertilizers, pesticides, and additives (Eick et al., 1999). In some industrial cities, urban soils are heavily polluted with Pb and the mean Pb concentration is much higher than its background value (Lu et al., 2003; Li et al., 2013).

Pb is easily absorbed by plants and accumulates in different tissues (Sharma and Dubey, 2005), then endangering human health through the food chain. Pb is toxic to many organ systems of the human body, such as the renal system, the cardiovascular system, the reproductive system, and the nervous system, leading to anemia, changes to sperm morphology and function, neurological disorders, renal tubular damage, a weakened immune system, hyperactivity, and enzyme changes (Gidlow, 2004; Liu et al., 2013). Therefore, Pb uptake, transport and accumulation in plants, particularly in edible parts, have attracted much attention (Liu et al., 2013). Although Pb is not an essential element for plants, many studies have indicated that some plants can absorb Pb through roots and translocate it to shoots (Tangahu et al., 2011; Liu et al., 2013; Wójcik and Tukiendorf, 2014). A large amount of lead was accumulated in roots, but only lower levels of Pb were translocated into shoots of water hyacinth seedlings (Malar et al., 2014). However, different plant species and plant organs may differ significantly in their ability to absorb, transport, and accumulate Pb (Alexander et al., 2006; Weng et al., 2009; Liu et al., 2010; Zhang et al., 2012). Furthermore, the accumulation and transport efficiency may vary within the same plant species depending on their genetic backgrounds (Su et al., 2014).

HM accumulation in plants is determined by many factors such as medium conditions (soil, nutrients, air), the uptake capacity of roots, the efficiency of translocation, and the distribution and redistribution of the metal among plant tissues (Zheng et al., 2011). It has been reported that differences in Pb translocation within plants may result from different chemical forms of Pb (Liu et al., 2013). The transport, toxicity and biological validity of HMs in plants are closely related to their subcellular distribution and chemical form of HMs in plants (Wang et al., 2008). Chemical speciation and distribution characteristics of HMs in plants also affect the uptake and tolerance of plants to HMs (Wang et al., 2008). In roots, Pb immobilization is mainly due to the complexing ability of histidine, and the transport to the upper plant organs is mainly attributed to Pb complexes with organic acids (Massaccesi et al., 2014). Patterns of subcellular distribution and chemical forms of HM in plant are important reflections of plant accumulation and detoxification processes (Wang et al., 2008; Xu et al., 2011). Chemical forms and subcellular distribution differ greatly among HMs, plant species, cultivars, and ecotypes (Wu et al., 2005; Wang et al., 2008; Xu et al., 2011). Pb taken up by roots can be deposited at many sites in the tissue (Inoue et al., 2013). Recent studies of root cells of *Pisum sativa* under high Pb stress have shown that Pb mainly accumulated on the cell wall, the cell membrane, cell voids, mitochondria, and peroxisomes (Malecka et al., 2008). A study of *Sedum alfredii* also found that Pb accumulated mainly on the cell wall and very little was found on the membrane fraction of roots, stem, and leaves (He et al., 2003).

Radish (*Raphanus sativus* L.), belonging to the Brassicaceae family, is an important vegetable crop worldwide, especially in East Asia. Recently, large phenotypic variations in root uptake and concentration of Pb among different tissues in radish plants were observed. Radish roots and hypocotyls accumulated 50% and 35% of the total lead content, respectively (Massaccesi et al., 2014). Pb particles were mainly deposited in the intercellular space, and only a small proportion was found in the cell wall of radish roots (El-Beltagi and Mohamed, 2010). However, another study indicated that Pb was deposited in the cell wall and vacuoles in lateral roots but not in the taproot (Inoue et al., 2013). To characterize the genotypic differences of Pb absorption as well as the accumulation, in our previous study, a hydroponic culture supplemented with Pb (NO_3_)_2_ was carried out for 36 radish genotypes which were selected based on their main traits including skin and flesh color, taproot shape, and size. The results showed that root Pb concentrations varied significantly among various radish genotypes and the concentration of the highest Pb accumulation genotype was about 7.5 times higher than that of the lowest one. Some high-accumulation genotypes including “NAU-XLM,” “NAU-CH,” and low-accumulation ones including “NAU-XHT” and “NAU-YH” were identified (Shen et al., 2015). Although Pb uptake by plant roots has been studied extensively, little is known about the translocation and distribution of Pb within radish plants. The aims of this study were to investigate: (1) the transport of Pb within intact radish plants under different Pb regimes; (2) subcellular distribution and chemical forms of Pb among various parts of radishes under different Pb regimes; and (3) Pb localization in radish root cells. The results revealed the characteristics of Pb uptake, transport, and distribution in radish, which may provide valuable information for better understanding of the molecular mechanism of Pb translocation in radish and for further development of low-Pb-content vegetable crops.

## Materials and methods

### Plant materials

Two advanced inbred lines of radish, “NAU-XLM” (high-Pb-accumulation) and “NAU-XHT” (low-Pb-accumulation), which were self-pollinated for more than 20 generations, were used for the study of Pb accumulation and transport as well as analysis of subcellular distribution and chemical forms of Pb. Additionally, the “NAU-XLM” was chosen to investigate Pb localization in root cells.

### Hydroponic experiment and plant sampling

Seeds of radish were firstly surface sterilized and then incubated at 25°C in the dark. The germinated seeds were sown in plastic pots containing soil and nursery substrate (soil: turf: vermiculite = 1:1:1) in a greenhouse. After 1 month, radish seedlings of similar size were selected and transferred to the modified half-strength Hoagland's nutrient solution (Wang et al., 2013). The solution was renewed every 4 days. A week later, seedlings of the two genotypes were treated with 0, 200 and 500 mg/L Pb(NO_3_)_2_ for 2, 4, 6, 8 days. Each treatment consisted of three replicates. For the amount of plant parts from one individual is not enough for digestion and Pb concentration measurement, the plant parts from three randomly selected plants of each replicate with five individuals were pooled to get sufficient amount of samples.

The plants were immersed in 0.02 mol/L Na_2_EDTA for 20 min, and then rinsed with deionized water. Thereafter, the plants of the NAU-XLM were separated into leaves, petioles, roots, and lateral roots (LR), whilst the plants of the NAU-XHT were separated into leaves, petioles, root heads (dwarf stem), root necks (the upper root that originates from the hypocotyl, without lateral roots), true roots (the lower root that consists of true root tissue), the skins of these parts and lateral roots. For each replicate, the same plant parts which had been obtained from the three selected individuals were pooled with the amount of about 2.0 g in fresh weight except the LR with amount of about 0.4 g. Samples were dried in an oven overnight at 70°C to constant weight and then ground into fine powder. And then approximately 0.2 g samples (~0.1 g for LR) were weighed with 5.0 mL mixture of concentrated HNO_3_–HClO_4_ (4:1, v/v), which were well-mixed and left to stand for at least 12 h at room temperature. The digestion was carried out in a heating block using the temperature program according the previous study by Zhao et al. (1994). The samples were evaporated to dryness with a final temperature of 180°C. After digestion and cooling, 10 ml of HNO_3_ (2.5% v/v) was added, which were mixed and rewarmed at 80°C for 30–60 min. For Atomic Absorption spectrophotometry (AAS) measurement, each treatment consisted of three replicates. Each replicate was measured three times.

### Tissue fractionation and chemical form extraction

For analysis of subcellular distribution and chemical forms of Pb, the plants were immersed in 0.02 mol/L Na_2_EDTA, rinsed with deionized water and separated into leaves, roots without skins, skins, and lateral roots. Fresh samples (0.5 g) were homogenized in pre-cooled extraction buffer containing 0.25 mol/L sucrose, 0.05 mol/L Tris-HCl (pH 7.5) and 0.001 mol/L C_4_H_10_O_2_SS_2_. Cells were separated into four fractions (F1–F4) with differential centrifugation technique (Weigel and Jäger, 1980; Zhou et al., 2008). The homogenate was centrifuged at 300 g for 30 s and the precipitation was designated as cell wall fraction (F1) mainly consisting of cell walls and cell wall debris, the resulting supernatant solution was further centrifuged at 2000 g for 15 min mainly consisting of chloroplasts and cell nuclei (F2), the supernatant solution was further centrifuged at 10,000 g for 20 min, referring to as mitochondrial components (F3), and soluble components containing ribosomes (F4, the supernatant), respectively. All steps were performed at 4°C.

The chemical forms of Pb were extracted using a sequence of different extractants (Xu et al., 1991; Xu and Wang, 2013): (1) 80% ethanol, extracting inorganic Pb including nitrate/nitrite, chloride; (2) Deionized water (d-H_2_O), extracting water-soluble Pb-organic acid complexes and Pb(H_2_PO_4_)_2_; (3) 1 mol/L NaCl, extracting pectates, protein integrated or adsorptive Pb; (4) 2% acetic acid (HAC), extracting undissolved lead phosphate including PbHPO_4_ and Pb_3_(PO_4_)_2_ and other Pb–phosphate complexes; (5) 0.6 mol/L HCl, extracting lead oxalate; and (6) Pb in residues. Fresh tissue (2 g) was cut into small pieces of 1–2 mm^2^ and transferred into a beaker with 25 ml extraction solution. Mixture was incubated at 30°C (about 17–18 h) and then the extraction solution was pooled. The residues were extracted again with the same extraction solution (25 ml) for another 2 h, which was repeated twice in the next 4 h. A total of 100 ml extraction solution was collected and evaporated to constant weight, and then digested with HNO_3_–HClO_4_ (4:1, v/v) for determination. After collection of the former extraction solution, the plant materials retained in the beaker were subjected to the next extractant with the similar procedures. For determination of Pb in residues, plant material was digested with HNO_3_–HClO_4_ (4:1, v/v) at the end of the sequential extraction. Lead concentrations associated with different chemical forms were determined by AAS (AAnalyst 700, Perkin-Elmer, USA) (Xu et al., 1991; Xu and Wang, 2013).

### Ultrastructural localization of Pb using transmission electron microscopy (TEM)

One to two millimeter (mm) sections of treated roots and lateral roots of the NAU-XLM were fixed in 2.5% glutaraldehyde (v/v) in 0.2 mol/L sodium phosphate buffer of pH 7.2 at 4°C for 12 h. The tissues were rinsed three times (1 h each time) in 0.2 mol/L phosphate buffer (pH 7.2). After that, the samples were post fixed in 1% (v/w) OsO_4_ for 2 h, then rinsed three times (1 h per time) in 0.2 M phosphate buffered solution (pH 7.2). Samples were dehydrated using a graded acetone and ethanol series (30, 50, 70, 80, and 90%), then the tissues were infiltrated and embedded in Spurr's resin and cut into ultrathin slices (80 nm) using a Power Tome–XL microtome. They were finally mounted on copper grids and observed using a transmission electron microscope (TEM, Hitachi H–7650), at an accelerating voltage of 80 keV (Lu et al., 2012).

### Pb concentration determination and data analysis

Pb concentration was determined using AAS (AAnalyst 700, Perkin-Elmer, USA) (Liu et al., 2010). The data obtained was analyzed with the statistical package of IBM SPSS Statistics v20.0 and EXCEL2010, and the graphs were produced with the EXCEL 2010.

## Results

### Pb uptake of in the high-Pb-accumulation line NAU-XLM

**Figure 1:**
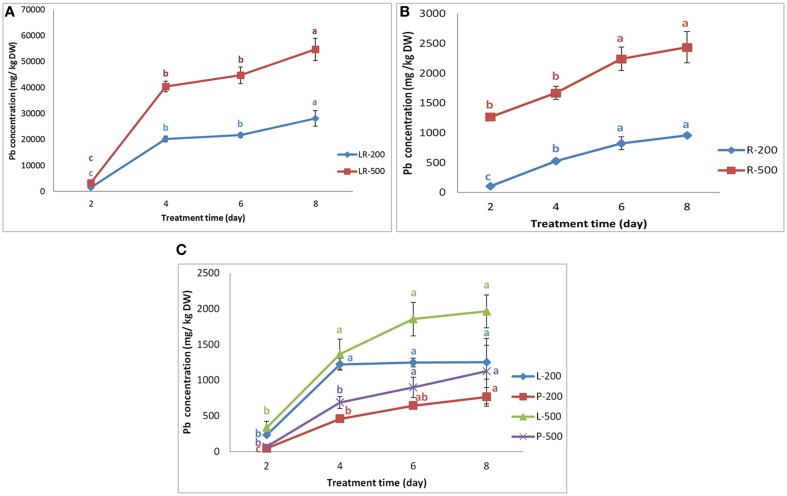
**Variation in the Pb concentration in different parts of NAU-XLM**. Panels **(A,B)** represent the Pb concentration in lateral roots (LR) and roots (R), respectively. LR-200 and R-200 was lateral roots and roots treated with 200 mg/L Pb(NO_3_)_2_; while LR-500 and R-500 was lateral roots and roots treated with 500 mg/L Pb(NO_3_)_2_. Panel **(C)** represents the Pb concentration in leaves (L) and petioles (P). L-200 and P-200 were leaves and petioles treated with 200 mg/L Pb(NO_3_)_2_; R-500 and P-500 were leaves and petioles treated with 500 mg/L Pb(NO_3_)_2_. Data are the mean ± SE (*n* = 3) and different letters (lowercase) indicate significant differences at *P* < 0.05.

In hydroponic culture conditions, as compared with control, no special obvious morphologic differences were found among individuals exposed to 200 mg/L Pb(NO_3_)_2_ for a maximum of 6 days, while the plants were hampered and grew abnormally when exposed to 500 mg/L Pb(NO_3_)_2_ after 6 days. Some plants were wilting and some mature leaves were rolling in chlorosis, and the root enlargement, and shoot elongation were relatively inhibited. The Pb concentration in each part of the radish increased with increasing Pb treatment duration and concentrations, but was always greatest in lateral roots (Supplementary Table 1). The results indicated that the NAU-XLM can accumulate large amounts of Pb in their tissues, for example, under a treatment of 500 mg/L Pb(NO_3_)_2_, the concentration of lead in roots increased from 1265.50 mg/kg on the second day to 2434.67 mg/kg on the eighth day. The Pb concentrations varied considerably among tissues. Under a treatment of 200 mg/L Pb(NO_3_)_2_, the distribution of Pb concentration among tissues followed the order of lateral roots >> leaves > roots > petioles; however, under 500 mg/L Pb (NO_3_)_2_, the order was lateral roots >> roots > leaves > petioles. Pb concentrations in lateral roots increased 11.8% and 11.3% under 200 mg/L and 500 mg/L treatments, respectively, from the second day to the fourth day, and then showed a small increase by 39.1 and 35.3%, respectively, in the next 4 days (Figure 1A). Root Pb concentrations showed a similar trend (Figure 1B). Pb concentrations in petioles had the largest increase, during the first 4 days, representing 9.0% and 8.5% under 200 and 500 mg/L Pb treatments, respectively, after which the rate of increase declined with a final increase by 19.1%, and 25% over the last 2 days (Figure 1C). A similar pattern appeared in Pb concentrations in the leaves under Pb treatments of 200 and 500 mg/L, but the scale of increase was smaller (Figure 1C).

### Pb uptake in the low-Pb-accumulation line NAU–XHT

When compared to their corresponding control, the results indicated that the NAU–XHT can also accumulate lots of Pb in their tissues (Supplementary Table 2). The Pb concentrations in tissues of the NAU–XHT varied considerably under treatment of 200 mg/L Pb (NO_3_)_2_. In general, Pb concentration in the tissues followed the order: lateral roots >> leaves > root heads > skin of true root > skin of root neck > petioles > true root flesh > root neck flesh. However, on the eighth day, the order of Pb concentration was lateral roots >> skin of true root > root heads > leaves > skin of root neck > petioles > true root flesh > root neck flesh. Under the treatment of 500 mg/L Pb (NO_3_)_2_, the order was lateral roots >> skin of true root > root heads > skin of root neck > leaves > petioles > true root flesh > root neck flesh, except on the second day, when the order was lateral roots >> root heads > skin of true root > skin of root neck > leaves > true root flesh > petioles > root neck flesh.

**Figure 2:**
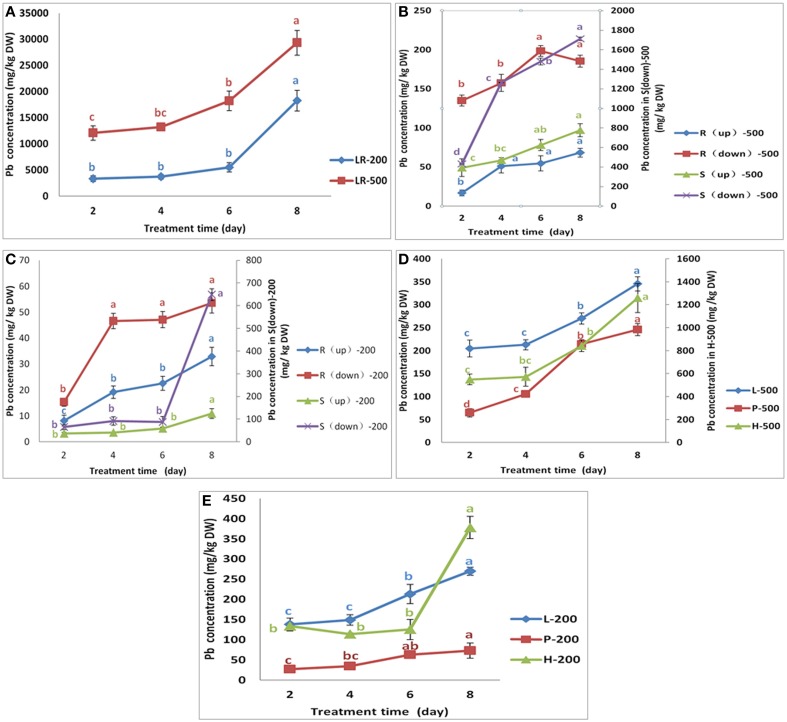
**Variation in the Pb concentration in different parts of NAU-XHT**. Panel **(A)** represents the Pb concentration in lateral roots (LR). LR-200 and LR-500 were lateral roots treated with 200 mg/L and 500 mg/L Pb(NO_3_)_2_, respectively. Panels **(B,C)** represent the Pb concentration in root flesh and skin(S) treated with 500 mg/L and 200 mg/L Pb(NO_3_)_2_, respectively. R(up)-500 and S(up)-500 were root neck and skin; R(down)-500 and S(down)-500 were true root and skin; R(up)-200 and S (up)-200 were root neck and skin; R(down)-200, and S(down)-200 were true root and skin. Panel **(D)** L-500, P-500, and H-500 were leaves (L), petioles (P), and root heads (H) treated with 500 mg/L Pb(NO_3_)_2_. Panel **(E)** L-200, P-200, and H-200 were leaves, petioles, and root heads treated with 200 mg/L Pb(NO_3_)_2_. Data are the mean ± SE (*n* = 3) and different letters (lowercase) indicate significant differences at *P* < 0.05.

The rate of increase in Pb uptake in lateral roots increased gradually, showing 9.5% and 10.8% increase from day 2 to day 4, and 61% and 230.7% increase from day 6 to day 8 under the Pb treatment of 200 mg/L and 500 mg/L, respectively (Figure 2A). The rate of Pb uptake in skins of true root decreased gradually, initially increasing by 193% from day 2 to day 4, and finally 15.7% from day 6 to day 8 under the 500 mg/L Pb treatment (Figure 2B). However, Pb concentrations in the skins of true roots increased by 40.8% from day 2 to day 4, and then decreased by 4.3% from day 4 to day 6, after which it increased by 637% from day 6 to day 8 under the 200 mg/L Pb treatment (Figure 2C). The rate of increase of Pb uptake in the skins of root necks increased gradually, initially by 9.3% from day 2 to day 4, and finally 110% from day 6 to day 8 under the 200 mg/L Pb treatment (Figure 2C). Pb concentrations in the skins of root necks increased over 19.5% at the first 4 days, by 33.2% in the next 2 days, after which there was a smaller increase (24.3%) under the 500 mg/L Pb treatment (Figure 2B). Pb concentrations in true roots increased by 204% during the first 4 days, and then by a much smaller increase of 1.2%, after which it increased by 13.8% under the 200 mg/L Pb treatment (Figure 2C).

Pb concentrations in root neck flesh showed a similar trend to that of true root flesh, although the scale of change was different (Figures 2B,C). However, the increased trend in the true roots under 500 mg/L Pb treatment was opposite of 200 mg/L Pb treatment (Figures 2B,C). Pb concentrations in the root head decreased by 15.1%, in the first 4 days, and then increased by 10.3% in the next 2 days, finally increasing by 202% in the last 2 days, under the 200 mg/L Pb treatment. However, under 500 mg/L Pb treatment, Pb concentrations in root head increased by 4.3% in the first 4 days, then by 47.2% in the next 2 days, and finally by 49.4% in the last 2 days. Pb concentration in petioles increased by 30.4% and 63.6% in the first 4 days, then by 81.59% and 103% in the next 2 days, and finally by 15.2% and 14.81% in the last 2 days under Pb treatments of 200 and 500 mg/L (Figures 2D,E). The concentration of Pb in leaves of the NAU–XHT under the 200 mg/L treatment showed a similar trend to that of petioles. However, the leaf Pb concentrations increased by 4.0% in the first 4 days, then by 27% in the next 2 days, and by 27.9% in the last 2 days under the 500 mg/L Pb treatment.

### Pb translocation in radish

Translocation factors (TFs) are usually used to evaluate the capacity of a plant to translocate HMs from underground to aboveground. There were large differences in the TFs between lines. TFs ranged from 0.049 to 0.172 in the NAU-XLM under Pb treatments of 200 and 500 mg/L (Table 1). The TFs of the NAU–XHT ranged from 0.018 to 0.048 under 200 and 500 mg/L Pb treatments (Table 1). Pb was mostly concentrated in the underground parts, and only a small amount of Pb was translocated to the aboveground parts. The TFs of the NAU-XHT under Pb treatments of 200 and 500 mg/L showed a similar trend to those of the NAU-XLM. The TFs of both lines decreased from the second day to the fourth day, and then increased from the fourth day to the sixth day before decreasing again in the last 2 days.

**Table 1:** Translocation factors (TFs) in four time periods under two Pb treatments^*^.

**Cultivars**		**NAU-XLM**		**NAU-XHT**	
**Treatment (mg/L)**		**200**	**500**	**200**	**500**
Leaf	2d	0.143 ± 0.016a^**^	0.074 ± 0.018a	0.039 ± 0.005a	0.015 ± 0.002a
	4d	0.060 ± 0.006b	0.032 ± 0.004b	0.038 ± 0.001a	0.013 ± 0.001a
	6d	0.056 ± 0.005b	0.040 ± 0.004b	0.037 ± 0.002a	0.013 ± 0.002a
	8d	0.046 ± 0.013b	0.035 ± 0.001b	0.014 ± 0.001b	0.010 ± 0.001a
Petiole	2d	0.029 ± 0.007a	0.016 ± 0.001a	0.008 ± 0.001ab	0.005 ± 0.000c
	4d	0.022 ± 0.000a	0.017 ± 0.002a	0.009 ± 0.001a	0.007 ± 0.000b
	6d	0.032 ± 0.003a	0.019 ± 0.003a	0.011 ± 0.002a	0.010 ± 0.001a
	8d	0.030 ± 0.007a	0.021 ± 0.010a	0.004 ± 0.001b	0.007 ± 0.001b
Above-ground	2d	0.172 ± 0.023a	0.090 ± 0.017a	0.047 ± 0.006a	0.020 ± 0.002a
	4d	0.082 ± 0.006b	0.049 ± 0.003b	0.043 ± 0.005a	0.020 ± 0.000a
	6d	0.085 ± 0.004b	0.059 ± 0.007ab	0.048 ± 0.004a	0.023 ± 0.002a
	8d	0.073 ± 0.020b	0.055 ± 0.008b	0.018 ± 0.002b	0.018 ± 0.002a

* TF = C_i_/C_r_, C_i_ is the Pb concentration in the aboveground tissues and C_r_ is the Pb concentration in the roots.

** Data are the means of three replicates (± SE). Different letters indicate significant differences according to Duncan's test at P < 0.05 (lowercase).

With increasing treatment concentration, the TFs of leaves and petioles in both lines showed a downward trend except on the eighth day when TFs increased. Under the 200 mg/L Pb treatment, the TFs of leaves in both lines showed a downward trend with increasing treatment duration. Under the 200 mg/L Pb treatment, the TFs of petioles in the NAU-XHT increased in the first few days and then declined slightly, the opposite trend to TFs in petioles of the NAU-XLM. Under the 500 mg/L Pb treatment, TFs in the leaves of the NAU-XLM showed a similar trend to that of TFs in the aboveground parts of the NAU-XLM, and the TFs rose in the petioles of both lines. This observation indicated that there were differences in the capacity to translocate Pb between lines, and that the Pb concentration in both underground and aboveground parts increased with increasing treatment concentration and duration, but the proportion of the increase between the two parts was different.

### Chemical forms of Pb within radish

**Figure 3:**
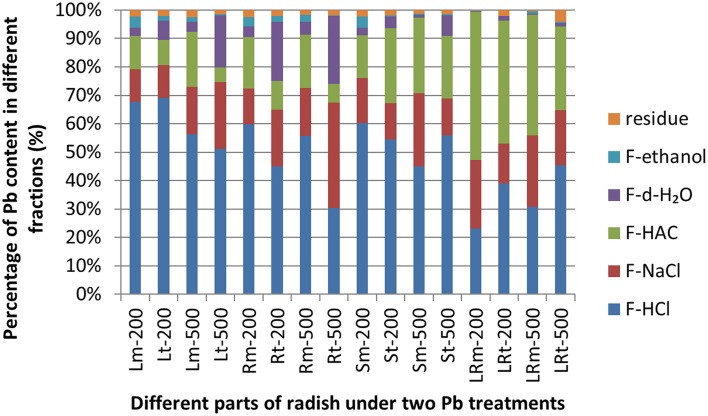
**Different chemical forms of Pb and its distribution in different tissues of radish**. Lm-200, Rm-200, Sm-200, and LRm-200 were leaves(L), roots(R), skins (S), and lateral roots(LR) of NAU-XLM treated with 200 mg/L Pb(NO_3_)_2_; Lm-500, Rm-500, Sm-500, and LRm-500 were leaves, roots, skins, and lateral roots of the NAU-XLM treated with 500 mg/L Pb(NO_3_)_2_; Lt-200, Rt-200, St-200, and LRt-200 were leaves, roots, skins, and lateral roots of the NAU-XHT treated with 200 mg/L Pb(NO_3_)_2_; Lt-500, Rt-500, St-500, and LRt-500 were leaves, roots, skins, and lateral roots of NAU-XHT treated with 500 mg/L Pb(NO_3_)_2_. Residue represents Pb concentration in residue; F-ethanol represents ethanol-extractable form, inorganic Pb including nitrate/nitrite, chloride; F-dH_2_O extracting water-soluble Pb of organic acid and Pb(PO_4_)_2_; F-HAC represents HAC- extractable form, undissolved lead phosphate including PbHPO_4_ and Pb_3_(PO_4_)_2_; F-NaCl extracting pectates, protein integrated Pb; F-HCl represents HCl- extractable form, extracting lead oxalic. All abbreviations ending with “m” refer to NAU-XLM and “t” to NAU-XHT, “R” to root and “LR' to lateral root, “S” to skin and “L” to leaf.

The Pb extracted by 2% HAC, 0.6 mol/L HCl, and 1 mol/L NaCl were the predominant chemical forms of Pb, representing 89.96–99.30% of the total Pb in different tissues of the NAU-XLM (Figure 3; Supplementary Table 3). In the lateral roots, the concentration of Pb extracted by 2% HAC was greatest (42.50–51.97%), followed by 1 mol/L NaCl and lastly 0.6 mol/L HCl; in the skins, the concentration of Pb extracted with 0.6 mol/L HCl was the greatest, representing 60.28% and 44.93% under Pb treatments of 200 and 500 mg/L, but the proportion of 2% HAC-extractable and 1 mol/L NaCl-extractable Pb became increasingly greater with increasing Pb treatment. In the roots and leaves of the NAU–XLM, the concentration of Pb extracted by 0.6 mol/L HCl was greatest (55.60–63.46% in roots and 56.32–67.71% in leaves (Figure 3). However, chemical forms of Pb extracted by d-H_2_O increased with enhancing Pb treatment strength (Figure 3). The Pb extracted by 2% HAC, 0.6 mol/L HCl, and 1 mol/L NaCl were the predominant chemical forms of Pb in all treatment levels in the lateral roots and skins of NAU-XHT, representing 94.21–96.23% (lateral roots) and 90.97–93.60% (skins) of the total Pb (Figure 3). In the roots and leaves of the NAU-XHT, Pb concentrations extracted by d-H_2_O enhanced with increased Pb treatment strength; in addition, the proportion of 0.6 mol/L HCl-extractable Pb, 1 mol/L NaCl-extractable Pb and d-H_2_O-extractable Pb was the highest, representing 85.84–92.98% of the total Pb (Figure 3). Moreover, Pb concentration in taproots without skin and aboveground increased with enhancing d-H_2_O extractable Pb concentration (Figure 3).

### Subcellular distribution of Pb in radish

**Figure 4:**
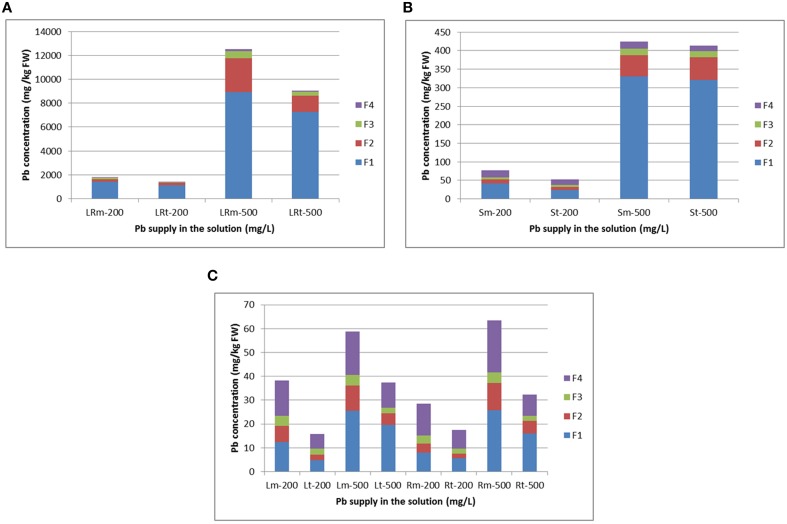
**Subcellular distribution of Pb and its stacking proportion in lateral roots (A), root skin (B), and leaves and roots (C) of radish**. LRm-200 and LRt-200 as well as LRm-500 and LRt-500 represent lateral root treated with 200 and 500 mg/L Pb(NO_3_)_2_, respectively; Sm-200 and St-200 as well as Sm-500 and St-500 represent root skin treated with 200 and 500 mg/L Pb(NO_3_)_2_, respectively. Lm-200 and Lt-200 as well as Rm-200 and Rt-200 represent leave and root flesh treated with 200 mg/L Pb(NO_3_)_2_, respectively; Lm-500 and Lt-500 as well as Rm-500 and Rt-500 represent leaves and roots treated with 500 mg/L Pb(NO_3_)_2_, respectively. F1, F2, F3, and F4 represent the fraction of cell wall, nucleus and chloroplasts, mitochondria, and soluble components containing ribosomes, respectively. All abbreviations ending with “m” refer to NAU-XLM and “t” to NAU-XHT, “R” to root, and “LR” to lateral root, “S” to skin and “L” to leaf.

Pb concentrations in the different subcellular components of various radish tissues increased with enhanced Pb treatment strength (Figure 4; Supplementary Table 4). In lateral roots, most Pb was present in the cell wall fraction and nucleus and chloroplasts fraction, representing 87.62–93.76% and 93.72–95.29% of Pb in the NAU-XLM and NAU-XHT, respectively (Figure 4A). In skins, a large proportion of Pb was bound to the cell wall fraction, representing 54.44–77.94% and 46.22–77.59% of Pb in the NAU-XLM and NAU-XHT, respectively (Figure 4B). However, under the 200 mg/L Pb treatment, a large amount of Pb also accumulated in soluble components containing ribosomes, representing 23.35% and 29.39% of total Pb in the skins of the NAU-XLM and NAU-XHT, respectively (Figure 4B). In roots, most Pb was localized in the cell wall fraction and soluble components containing ribosomes, representing 74.55–74.89% and 76.52–77.10% of Pb, in the NAU-XLM and NAU-XHT, respectively. The proportion of Pb in the cell wall fraction increased, but the proportion in soluble components containing ribosomes decreased with enhancing Pb treatment strength (Figure 4C). A similar result was found in leaves, the proportions of Pb in the cell wall fraction and soluble components containing ribosomes were 71.17–74.61% and 69.05–80.46% in the NAU-XLM and NAU-XHT, respectively (Figure 4C). A greater proportion of Pb was bound to the cell wall fraction in the NAU-XHT than that in the NAU-XLM, and a smaller proportion of Pb was localized in the soluble fraction of the NAU-XHT than that in the NAU-XLM.

### Ultrastructural localization of Pb in cells of radish

**Figure 5:**
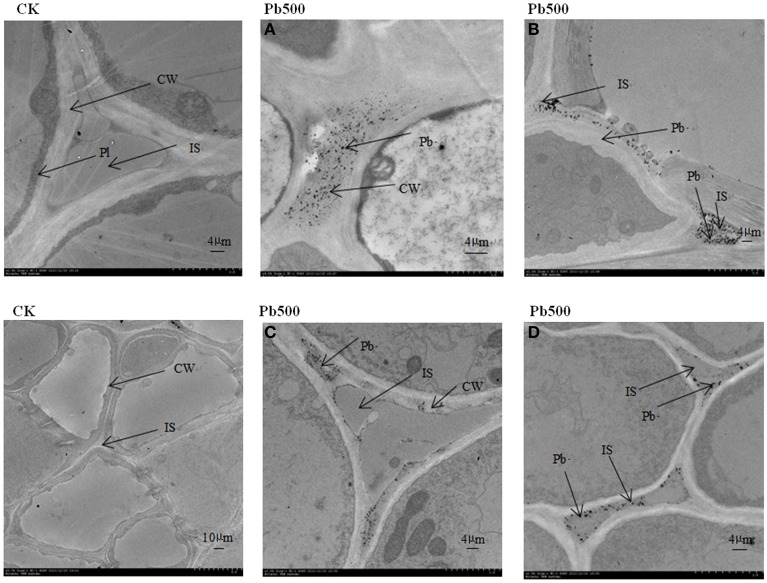
**Ultrastructure of transverse sections of lateral roots in NAU-XLM under 500 mg/L Pb treatment**. Electron-dense particles were localized in CW, cell wall **(A,C)** and IS, intercellular space **(B,D)**. Pl refers to plasmalemma.

**Figure 6:**
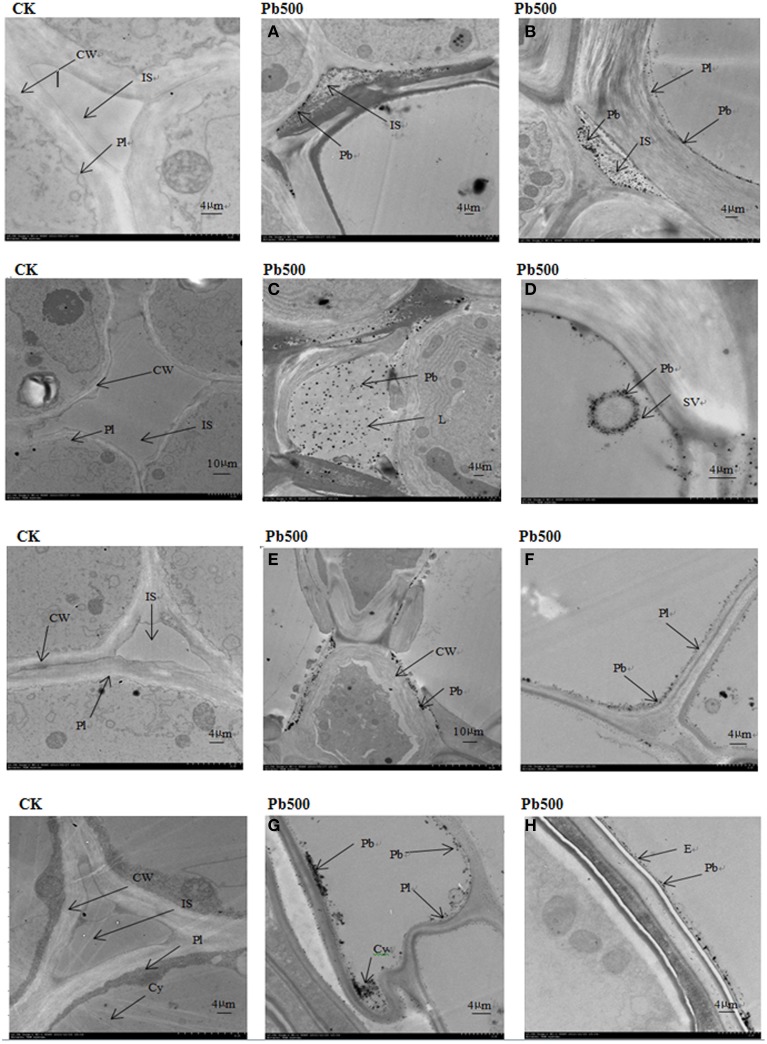
**Ultrastructure of transverse sections of roots in NAU-XLM under 500 mg/L Pb treatment**. Electron-dense particles were localized in IS, intercellular space **(A,B)**; L, lumen **(C)**; SV, small vacuole **(D)**; CW, cell wall **(E)**; Pl, plasmalemma **(F)**; Cy, cytoplasm **(G)**; and E, epidermis **(H)**.

To show the Pb particles in subcellular fractions visibly, the roots and lateral roots of NAU-XLM under the 500 mg/L Pb treatment were sampled for TEM analysis. Transverse sections of radish roots and lateral roots observed by TEM revealed many Pb particles in cell walls (Figures 5A,C, 6E) and in the intercellular space (Figures 5B–D,6A,B) of both taproot and lateral root cells. Large amounts of Pb precipitates were distributed mainly around the surface of the vacuole membrane (Figure 6D) and lumen (Figure 6C). Precipitates of Pb were also found in the plasma lemma (Figures 6B, F), cytoplasm (Figure 6G), and epidermis (Figure 6H).

## Discussion

### Uptake and transport of Pb in radish

Recently, with increasing concern about the HM pollution and safe vegetable production, there have been many studies on the uptake, accumulation, and translocation of HMs in vegetable crops. Radish, an important vegetable crop, can uptake and accumulate HMs including Pb in the root (El-Beltagi and Mohamed, 2010; Inoue et al., 2013). Our study indicated that the lateral roots accumulated a large proportion of Pb, representing 66.29–91.56%, and 82.35–92.04% of Pb uptake in the NAU-XLM and NAU-XHT, respectively. The results also show that lateral roots of the NAU-XLM are enriched with a large amount of Pb when they are exposured to Pb in the solution for the first time (Figure 1A), then the rate of Pb enrichment decreased, after which there is an increase again with increasing treatment duration. However, compared with the NAU-XLM, the uptake of Pb in lateral roots of the NAU-XHT was smaller for the first stage, and then the rate of Pb enrichment increased gradually with increasing treatment duration (Figure 2A). The skins accumulated a relatively larger portion of Pb (2.40–10.80%) than that of taproots without the skins (0.44–1.56%) in the NAU-XHT. Moreover, the skins with lateral roots accumulated more Pb (1.44–7.88%) than the smooth skins without lateral roots (0.63–2.92%).

The root heads of radish also accumulated high levels of Pb (1.90–3.95%) in the NAU-XHT. Similar to many other studies (Liu et al., 2003; El-Beltagi and Mohamed, 2010; Inoue et al., 2013), most Pb was retained in underground tissues, and little was transported to aboveground parts, as demonstrated by TFs. The Pb in aboveground parts may have been absorbed from the treatment solution or it may have been transported from underground parts. Generally, the Pb concentration in leaves was greater than that in petioles under Pb treatments of 200 and 500 mg/L, but the Pb concentration in petioles increased faster than in leaves with an increase in treatment duration and concentration. Previous studies have demonstrated that metal-tolerant plants accumulated higher concentrations of HMs in roots and lower concentrations in shoots compared with the non-metal-tolerant plants (Xu et al., 2011; Lu et al., 2014). Our results also indicated that this was an important tolerance mechanism of radish. In this study, radish lateral roots initially took up a large amount of Pb, and then a portion of the Pb was absorbed and a little was transported upward. Radish skins accumulated a lot of Pb, while roots without skin showed minimum uptake of Pb.

### Chemical forms of lead in radish

Different chemical forms affect the degree of toxicity and migration of HMs in plants (Fu et al., 2011; Weng et al., 2012). For instance, water-soluble Pb in inorganic form (extracted by 80% ethanol) and in organic form (extracted by d-H_2_O) exhibited higher chances of translocation. In these forms, Pb can easily penetrate into the cytoplasm and attach to the cell organelles as well as intercellular spaces in soluble fraction, having a more harmful effect on plant cells in comparison with the undissolved Pb–phosphate (extracted by 2% HAC) and Pb oxalate (extracted by 0.6 mol/L HCl) (Xu et al., 2011). The majority of Pb in radish tissues was integrated undissolved Pb oxalate (extracted by 0.6 mol/L HCl), protein and pectates (extracted by 1 mol/L NaCl) and Pb–phosphate complexes (extracted by 2% HAC). Moreover, in various tissues under Pb treatment, the NAU-XHT, a relatively low accumulation genotype, had a higher proportion of Pb in water-soluble forms, and a lower proportion (74.11–96.24 %) of undissolved Pb oxalate, protein and pectates, and Pb–phosphate complexes forms than the NAU-XLM (89.96–99.30%), a relatively high accumulation genotype. The higher concentration of undissolved Pb phosphate, pectates and protein integrated Pb and lower concentration of water–soluble Pb in the underground parts in plants may be a possible mechanism of tolerance to Pb toxicity in these relatively Pb–tolerant genotypes.

### Subcellular distribution and location of lead in radish

Cell compartmentalization and complexity, including cell wall deposition and vacuolar compartmentation play a significant role in HM detoxification, tolerance, and hyperaccumulation in plants (Wang et al., 2008; Fu et al., 2011). The cell wall is the first barrier preventing HMs from damaging plant cells. It contains mainly pectic acid, polysaccharides, and protein and provides plentiful HM ion exchange sites, which can fix Pb ions and restrict their movement across the cytomembrane (Inoue et al., 2013). In addition, Pb^2+^ binds to carboxyl groups which are possibly pectin compounds of cell walls (Inoue et al., 2013). The cell wall is the main binding site for various HM ions in plant species (Xu et al., 2012; Hou et al., 2013; Lu et al., 2014). However, when cell wall binding sites reach saturation, most intracellular HM ions are transported to vacuoles, and then chelated with citric acid, oxalic acid, and other organic acids, through which the HM ions are segregated (Kramer, 2000; Weng et al., 2012). In this study, a large portion of Pb was found to be stored in the cell wall fraction (32.07–80.40%). Moreover, ultrastructural observation also revealed plentiful Pb deposits on the cell wall of roots and lateral roots. The soluble fraction, containing mainly vacuoles is the subdominant HM binding site.

We found a significant percentage of Pb stored in soluble components in the leaves and roots of both lines. Moreover, a higher proportion of Pb was bound on the cell wall fraction of the NAU-XHT than that of the NAU-XLM, and a lower proportion of Pb was localized in the soluble fraction of the NAU-XHT than that of the NAU-XLM. This study also indicated that a relatively high proportion of the cell wall fraction could prevent Pb from penetrating into the cell interior, resulting in the reduction of Pb concentration in soluble fractions. Qiu et al. (2011) also found that the cadmium (Cd) compartment functioned better in a low-Cd-accumulating cultivar compared with a high-Cd-accumulating cultivar of flowering cabbage. Previous studies have also showed that the proportion of Cd associated with organelles was much greater in leaves than in roots and stems, which probably result from the preferential accumulation of Cd in chloroplasts (Ramos et al., 2002; Wang et al., 2008). However, similar results were not found for *Kandelia obovata* (Weng et al., 2012) and in this study. Ultrastructural observation of roots and lateral roots also indicated Pb deposited in intercellular spaces, on cell walls, in plasma lemma, lumina, and vacuoles, which was in agreement with previous studies (Malecka et al., 2008; El-Beltagi and Mohamed, 2010; Inoue et al., 2013).

**Figure 7:**
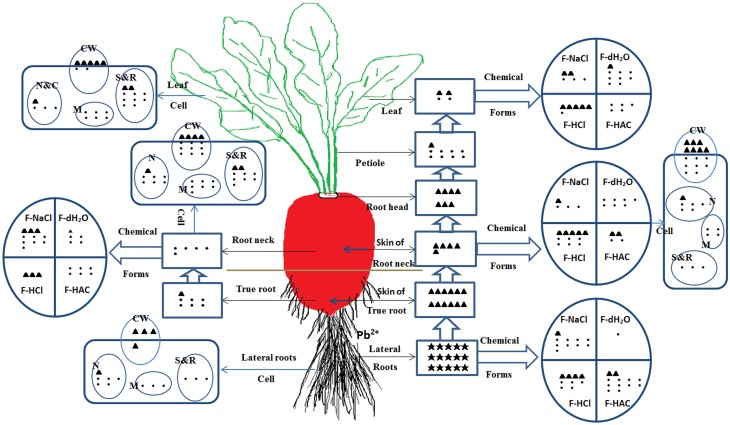
**The uptake, translocation and distribution of Pb in radish line NAU–XHT under 500 mg/L Pb treatment**. Symbol “•” represents basic concentration unit, “▲” represents 10-fold of the unit, “★” represents 100-fold of the unit. The number of unit symbol refers to Pb relative concentration for tissue levels, chemical forms, and subcellular fractions from various plant parts. F-HCl represents HCl-extractable form, lead oxalic; F-HAC represents HAC-extractable form, undissolved lead phosphate including PbHPO_4_ and Pb_3_(PO_4_)_2_; F-NaCl extracting pectates, protein integrated Pb; F-dH_2_O extracting water-soluble Pb of organic acid, and Pb(PO_4_)_2_. CW (Cell wall), N (Nucleus), M (Mitochondria), N&C (Nucleus and Chloroplasts fraction), S&R (Soluble fraction and Ribosome).

With the development of industry and the expansion of population, the anthropogenic Pb in soils has become one of the most toxic HMs, which may cause potential health risks to human beings and animals through the food chain. Therefore, minimizing the intake of Pb from vegetable crops and reducing Pb risks to human health is becoming a major worldwide ecological concern. In the present study, to investigate the mechanism of Pb accumulation and translocation in radish plant responding to the Pb stress, we selected the hydroponic culture, which allows us to control the environmental and stress conditions easily and effectively. HM accumulation in plants varied with plant species and genotypes, types of HMs, the uptake capacity and translocation efficiency of roots, and growth medium conditions (soil and nutrition solution) (Liu et al., 2010; Zheng et al., 2011). Therefore, further studies including field-culture experiments with combined HM treatments in soils could be carried out to illustrate the mechanism of HM accumulation for safe vegetable production. In this study, we characterized Pb transport, ultrastructural localization, and distribution of chemical forms of Pb in different tissues of radish plants with hydroponic culture (Figure 7). The results indicated relatively low TFs and high tolerance to Pb in radish. Most Pb was retained in lateral roots and skins thus restricting translocation to inner parts of the taproot and aboveground plant tissues. When the Pb was transported to inner tissues, it was mostly associated with undissolved chemical forms which have minimal motility. At a subcellular level, a large amount of Pb was deposited in intercellular space, bound to cell walls and stored in vacuoles, thereby reducing free Pb ions in the cytoplasm. These results could provide a solid foundation for further dissecting the mechanism of Pb accumulation, and facilitating development of low-Pb-content cultivar in root vegetable crops.

### Conflict of interest statement

The authors declare that the research was conducted in the absence of any commercial or financial relationships that could be construed as a potential conflict of interest.
