# Supplementary material for: Transport, ultrastructural localization, and distribution of chemical forms of lead in radish (Raphanus sativus L.)
Source: Front Plant Sci. 2015 May 8;6:293. doi: 10.3389/fpls.2015.00293 (PMC4424845; doi:10.3389/fpls.2015.00293)
Supplement: Supplementary file 1 [file Table1.DOC]

**Supplementary Table 1 Pb concentration in various tissues of NAU-XLM**

| Day | Concentration(mg/L) | Leaf  （mg/kg） | Petiole  （mg/kg） | Root  （mg/kg） | Lateral root  （mg/kg） |
| --- | --- | --- | --- | --- | --- |
| 2 | 0 | 3.83±0.18 | 1.15±0.13 | 1.36±0.07 | 14.10±1.42 |
|  | 200 | 234.70±8.20 | 46.15±4.76 | 107.08±11.20 | 1586.97±248.95 |
|  | 500 | 338.80±87.90 | 72.48±8.49 | 1265.50±48.50 | 3297.00±317.00 |
| 4 | 0 | 6.17±0.45 | 1.48±0.20 | 2.85±0.33 | 25.54±1.18 |
|  | 200 | 1224.67±82.18 | 459.80±33.00 | 527.73±38.20 | 20235.00±1135.00 |
|  | 500 | 1367.83±208.73 | 691.70±83.40 | 1669.50±110.50 | 40425.00±1995.00 |
| 6 | 0 | 13.21±1.74 | 5.80±0.26 | 2.91±0.21 | 23.90±1.98 |
|  | 200 | 1249.50±59.50 | 645.00±46.46 | 824.30±109.08 | 21670.00±950.00 |
|  | 500 | 1860.00±235.00 | 902.50±144.10 | 2241.50±195.50 | 44726.67±3187.09 |
| 8 | 0 | 10.29±0.30 | 5.68±0.29 | 5.77±0.42 | 35.48±1.61 |
|  | 200 | 1254.40±237.60 | 768.30±129.60 | 958.85±32.65 | 28140.00±2986.64 |
|  | 500 | 1966.50±229.50 | 1128.10±459.10 | 2434.67±261.17 | 54700.00±4291.34 |

**Supplementary Table 2** Pb concentration in various tissues of NAU-XHT

| Day | Concentration  (mg/L) | Leaf  （mg/kg） | Petiole  （mg/kg） | Root head  （mg/kg） | Root neck  （mg/kg） | True root  （mg/kg） | Skin of  root neck  （mg/kg） | Skin of  true root  （mg/kg） | Lateral root  （mg/kg） |
| --- | --- | --- | --- | --- | --- | --- | --- | --- | --- |
| 2 | 0 | 0.90±0.04 | 0.67±0.04 | 2.18±0.15 | 0.34±0.03 | 0.84±0.05 | 1.16±0.11 | 2.52±0.09 | 2.87±0.31 |
|  | 200 | 137.80±16.06 | 26.66±0.46 | 133.70±7.16 | 8.15±2.09 | 15.31±1.53 | 37.03±5.03 | 65.31±10.86 | 3364.00±559.00 |
|  | 500 | 204.60±18.05 | 64.60±8.79 | 548.90±46.93 | 16.81±3.62 | 135.10±7.20 | 393.00±90.76 | 432.80±3.90 | 12086.00±1398.96 |
| 4 | 0 | 2.05±0.27 | 1.03±0.08 | 2.27±0.35 | 0.30±0.02 | 1.04±0.13 | 1.59±0.057 | 1.38±0.23 | 3.64±0.22 |
|  | 200 | 148.80±12.85 | 34.77±3.71 | 113.50±3.55 | 19.15±2.36 | 46.56±2.96 | 40.49±0.99 | 91.96±18.47 | 3727.00±470.29 |
|  | 500 | 212.70±11.00 | 105.70±5.60 | 572.40±81.89 | 51.01±8.45 | 157.70±11.02 | 469.65±25.25 | 1266.00±3.00 | 13230.00±254.82 |
| 6 | 0 | 5.69±0.62 | 1.07±0.097 | 2.92±0.24 | 0.82±0.05 | 1.14±0.059 | 1.98±0.11 | 2.40±0.18 | 5.64±0.49 |
|  | 200 | 213.40±23.60 | 63.14±3.19 | 125.16±24.88 | 22.54±2.69 | 47.10±3.12 | 59.33±4.48 | 88.05±23.16 | 5528.00±903.00 |
|  | 500 | 270.10±12.07 | 214.10±4.90 | 842.30±51.45 | 54.73±9.62 | 198.70±6.90 | 625.50±57.61 | 1481.70±34.76 | 18236.60±1883.23 |
| 8 | 0 | 6.63±0.90 | 1.59±1.06 | 7.68±0.33 | 0.63±0.02 | 0.99±0.15 | 1.07±0.074 | 2.57±0.33 | 5.40±0.77 |
|  | 200 | 269.88±9.94 | 72.74±18.97 | 378.00±27.36 | 32.86±3.56 | 53.60±3.90 | 124.40±21.10 | 648.60±26.60 | 18280.00±1975.71 |
|  | 500 | 345.40±15.50 | 245.80±13.00 | 1258.00±126 | 68.44±5.67 | 185.60±7.55 | 777.30±65.70 | 1713.67±18.00 | 29353.00±2361.05 |

**Supplementary Table 3 Pb concentration in various chemical forms of radish**

| Tissues | F-Ethanol  （mg/kg） | d-H_2_O  （mg/kg） | NaCl  （mg/kg） | HAC  （mg/kg） | HCl  （mg/kg） | Residue  （mg/kg） |
| --- | --- | --- | --- | --- | --- | --- |
| Lm-200 | 3.58±0.31 | 2.41±0.11 | 10.27±0.41 | 10.44±0.17 | 60.19±2.31 | 1.99±0.08 |
| Lt-200 | 0.76±0.06 | 3.25±0.003 | 5.39±0.04 | 4.12±0.03 | 32.28±0.21 | 0.94±0.07 |
| Lm-500 | 2.03±0.07 | 4.62±0.41 | 21.14±2.10 | 24.28±2.21 | 71.17±6.24 | 3.13±0.06 |
| Lt-500 | 0.35±0.02 | 17.7±1.67 | 22.47±2.01 | 5.03±0.46 | 49.15±0.31 | 1.37±0.05 |
| Rm-200 | 2.78±0.145 | 3.16±0.02 | 10.75±0.09 | 15.52±1.45 | 50.95±4.12 | 2.12±0.17 |
| Rt-200 | 1.12±0.009 | 11.27±0.11 | 10.85±0.08 | 5.39±0.38 | 24.28±2.12 | 1.13±0.008 |
| Rm-500 | 2.75±0.13 | 5.36±0.37 | 19.83±1.67 | 21.97±1.35 | 65.01±1.75 | 1.99±0.16 |
| Rt-500 | 0.15±0.01 | 20.64±0.18 | 32.18±2.87 | 5.69±0.38 | 26.21±2.52 | 1.59±0.12 |
| Sm-200 | 5.58±0.27 | 3.88±0.29 | 22.92±1.94 | 22.04±1.76 | 87.75±7.35 | 3.38±0.08 |
| St-200 | 1.98±0.17 | 16.27±1.58 | 50.38±4.34 | 104.67±9.94 | 216.50±20.43 | 7.167±0.67 |
| Sm-500 | 3.11±0.02 | 5.48±0.36 | 147.17±11.34 | 152.37±11.56 | 257.10±13.64 | 7.02±0.02 |
| St-500 | 1.92±0,007 | 30.30±2.45 | 55.66±3.76 | 94.74±5.96 | 240.63±13.14 | 6.58±0,03 |
| LRm-200 | 16.85±1.35 | 10.07±0.09 | 1325±12.76 | 2839±19.79 | 260.97±2.45 | 11.32±1.03 |
| LRt-200 | 1.49±0.11 | 68.32±5.34 | 561.95±11.34 | 1746.50±95.45 | 1580.00±16.35 | 82.24±6.47 |
| LRm-500 | 155.95±13.78 | 48.57±4.77 | 4629.33±224.36 | 7828.67±118.64 | 5673.67±45.78 | 85.13±6.55 |
| LRt-500 | 15.77±1.23 | 88.59±7.65 | 1284.00±86 | 1959.00±61.92 | 3023.00±19.19 | 280.53±14.06 |

Note: Lm-200, Rm-200, Sm-200 and LRm-200 were leaves, roots, skins and lateral roots of ‘NAU-XLM’ treated with 200mg/L Pb(NO_3_)_2_; Lm-500, Rm-500, Sm-500 and LRm-500 were leaves(L), roots(R), skins (S) and lateral roots(LR) of ‘NAU-XLM’ treated with 500mg/L Pb(NO_3_)_2_; Lt-200, Rt-200, St-200 and LRt-200 were leaves, roots, skins and lateral roots of ‘NAU-XHT’ treated with 200mg/L Pb(NO_3_)_2_; Lt-500, Rt-500, St-500 and LRt-500 were leaves, roots, skins and lateral roots of ‘NAU-XHT’ treated with 500mg/L Pb(NO_3_)_2_. All abbreviations ending with ‘m’ refer to NAU-XLM and ‘t’ to NAU-XHT, ‘R’ to root and ‘LR’ to lateral root, ‘S’ to skin and ‘L’ to leaf.

**Supplementary Table 4 Pb concentration in subcellular fractions of radish**

| Tissues | F1（mg/kg） | F2（mg/kg） | F3（mg/kg） | F4（mg/kg） |
| --- | --- | --- | --- | --- |
| Lm-200 | 12.41±0.36 | 6.68±0.035 | 4.32±0.02 | 14.75±0.07 |
| Lt-200 | 4.71±0.38 | 2.33±0.17 | 2.57±0.11 | 6.24±0.53 |
| Lm-500 | 25.63±0.53 | 10.48±0.50 | 4.45±0.04 | 18.24±0.09 |
| Lt-500 | 19.52±0.50 | 5.04±0.11 | 2.27±0.16 | 10.62±0.06 |
| Rm-200 | 7.99±0.64 | 3.75±0.23 | 3.50±0.33 | 13.26±1.23 |
| Rt-200 | 5.61±0.48 | 2.86±0.23 | 2.15±0.06 | 7.87±0.08 |
| Rm-500 | 25.73±2.12 | 11.41±0.89 | 4.49±0.21 | 21.73±0.08 |
| Rt-500 | 15.93±1.06 | 5.43±0.51 | 2.18±0.16 | 8.86±0.63 |
| Sm-200 | 41.61±0.57 | 11.49±0.99 | 5.47±0.38 | 17.85±0.08 |
| St-200 | 24.40±2.20 | 8.21±0.09 | 4.66±0.39 | 15.52±0.58 |
| Sm-500 | 330.80±21.98 | 56.83±3.95 | 17.35±1.64 | 19.43±0.13 |
| St-500 | 320.80±4.07 | 61.31±0.86 | 16.50±0.29 | 14.81±1.41 |
| LRm-200 | 1427.00±6.36 | 224.45±19.02 | 105.25±4.69 | 38.24±0.09 |
| LRt-200 | 1123.50±54.85 | 202.53±2.33 | 60.88±0.61 | 28.04±1.97 |
| LRm-500 | 8904.33±155.33 | 2840.67±69.90 | 623.00±21.04 | 158.93±1.80 |
| LRt-500 | 7286.33±138.28 | 1349.67±59.09 | 336.70±6.36 | 90.04±0.91 |

Note: Lm-200, Rm-200, Sm-200 and LRm-200 were leaves, roots, skins and lateral roots of ‘NAU-XLM’ treated with 200mg/L Pb(NO_3_)_2_; Lm-500, Rm-500, Sm-500 and LRm-500 were leaves(L), roots(R), skins (S) and lateral roots(LR) of ‘NAU-XLM’ treated with 500mg/L Pb(NO_3_)_2_; Lt-200, Rt-200, St-200 and LRt-200 were leaves, roots, skins and lateral roots of ‘NAU-XHT’ treated with 200mg/L Pb(NO_3_)_2_; Lt-500, Rt-500, St-500 and LRt-500 were leaves, roots, skins and lateral roots of ‘NAU-XHT’ treated with 500mg/L Pb(NO_3_)_2_. F1 was cell wall fraction; F2 was nucleus and chloroplasts fraction; F3 was mitochondria fraction; F4 was soluble components containing ribosomes. All abbreviations ending with ‘m’ refer to NAU-XLM and ‘t’ to NAU-XHT, ‘R’ to root and ‘LR’ to lateral root, ‘S’ to skin and ‘L’ to leaf.
